# Genome-wide polymorphism and genic selection in feral and domesticated lineages of *Cannabis sativa*

**DOI:** 10.1093/g3journal/jkac209

**Published:** 2022-08-26

**Authors:** Patrick Woods, Nicholas Price, Paul Matthews, John K McKay

**Affiliations:** Graduate Degree Program in Ecology, Colorado State University, Fort Collins, CO 80523, USA; Department of Agricultural Biology, Colorado State University, Fort Collins, CO 80523, USA; Department of Agricultural Biology, Colorado State University, Fort Collins, CO 80523, USA; Bioinformatics Research Laboratory, Department of Biological Sciences, University of Cyprus, Nicosia, Cyprus; Graduate Degree Program in Ecology, Colorado State University, Fort Collins, CO 80523, USA; Department of Agricultural Biology, Colorado State University, Fort Collins, CO 80523, USA

**Keywords:** population structure, positive selection, balancing selection, Plant Genetics and Genomics

## Abstract

A comprehensive understanding of the degree to which genomic variation is maintained by selection vs. drift and gene flow is lacking in many important species such as *Cannabis sativa*, one of the oldest known crops to be cultivated by humans worldwide. We generated whole genome resequencing data across diverse samples of feralized (escaped domesticated lineages) and domesticated lineages of *C. sativa*. We performed analyses to examine population structure, and genome-wide scans for *F*_ST_, balancing selection, and positive selection. Our analyses identified evidence for subpopulation structure and further supported the Asian origin hypothesis of this species. Feral plants sourced from the United States exhibited broad regions on chromosomes 4 and 10 with high $\bar{F}$_ST_, which may indicate chromosomal inversions maintained at high frequency in this subpopulation. Both our balancing and positive selection analyses identified loci that may reflect differential selection for traits favored by natural selection and artificial selection in feral vs. domesticated subpopulations. In the US feral subpopulation, we found 6 loci related to stress response under balancing selection and 1 gene involved in disease resistance under positive selection, suggesting local adaptation to new climates and biotic interactions. In the marijuana subpopulation, we identified the gene *SMALLER TRICHOMES WITH VARIABLE BRANCHES 2* to be under positive selection, which suggests artificial selection for increased tetrahydrocannabinol yield. Overall, the data generated and results obtained from our study help to form a better understanding of the evolutionary history in *C. sativa*.

## Introduction

A central goal of evolutionary genetics is to understand the relative roles of recombination, selection, and drift in maintaining genetic variation in populations (Charlesworth and Charlesworth 2010). Since Charles Darwin first described the process of natural selection (Darwin and Kebler 1859), identifying the differences of how evolution proceeds under natural *vs.* artificial selection (domestication) has long intrigued scientists. Domestication of a species is associated with a “domestication syndrome” whereby adaptive genetic variation that enhances survival traits in nature is often selected against to favor genetics that enhance traits for human use (Brown 2010; Larson *et al.* 2014). Resequencing technology has been invaluable for understanding how the domestication syndrome affects genomic variation in crops by enabling scientists to compare entire genomes between domesticated lineages and their wild progenitors, which has revealed beneficial and deleterious mutations (Mace *et al.* 2013; Qin *et al.* 2014). Less understood is how genomic variation is affected when domesticated crop lineages escape farms to become feralized and revert to evolving under natural selection (Scossa and Fernie 2021). Species with both domesticated varieties and feralized crop populations are ideal systems to study these outstanding questions: which genes underlie adaptation under feralization vs. domestication and does adaptation under feralization vs. domestication occur through fixation of standing variation or through new mutations (Scossa and Fernie 2021)?

*Cannabis sativa* is one of the oldest known crops to be cultivated by humans for fiber, grain, and secondary metabolites such as terpenes and cannabinoids (Laursen 2015). From a scientific standpoint, *C. sativa* is interesting because it evolved dioecy and an annual life history from ancestors that were monoecious and perennial (Kovalchuk *et al.* 2020). As a result of extensive human vectoring and hybridization, the evolutionary history of this species remains convoluted. This domesticated species moved around with humans for thousands of years but then became prohibited in much of the world in 1937 due to the Marijuana Tax Act. Despite its prohibition, in the United States, it is estimated that 400,000 acres of hemp biomass were produced for textile and feedstock between 1942 and 1945 (West 1998). This created the current feralized populations in the United States that have been evolving under natural selection for decades (West 1998). As time has progressed, global regulations surrounding the legality of *C. sativa* have increasingly relaxed, which enables use of this species to better understand the evolutionary genetics of crop feralization.

Recent progress has been made in understanding the genomic variation of *C. sativa* and has identified several areas remain that require additional investigation (Kovalchuk *et al.* 2020; Ren *et al.* 2021). These areas include: (1) characterization of the genomic variation in broader samples such as those maintained by the Institute of Plant Genetics (IPK), (2) formal testing of the out of Asia hypothesis, and (3) comparison of balancing and positive selection patterns between domesticated and feralized subpopulations to better understand the genetics underlying their domestication/loss of domestication syndrome and local adaptation. Addressing these 3 areas is necessary for a more comprehensive characterization of this species’ evolutionary history.

In this study, the genomic variation of ∼80 previously unexamined *C. sativa* accessions, including industrial hemp varieties, feral populations in the United States, and accessions from the IPK Gatersleben seed bank, were characterized using WGS. Several publicly available WGS datasets for additional accessions data were also incorporated into our analyses for a more comprehensive study. Our study identified results consistent with previous reports while also expanding upon the current knowledge of *C. sativa* genomics with new discoveries. Through our analyses, we identify additional evidence for subpopulation structure, further support for the Asian origin of *C. sativa*, and find evidence for both balancing and positive selection on loci that potentially reflect the domestication/loss of domestication syndrome and local adaptation of cultivated and feral subpopulations.

## Materials and methods

### Sample collection

Seeds representing 20 industrial hemp cultivars were imported to Colorado for a set of field trials (Campbell *et al.* 2019). Additional seeds representing 51 *C. sativa* accessions stored at the IPK Gatersleben germplasm bank were imported from Germany to Colorado. Four seeds from each accession were sown in 3″ × 3″ pots in the Colorado State University greenhouse, and leaf samples were harvested for DNA extraction once the plants were well into their vegetative stage.

We also collected leaf tissue from feral *C. sativa* population located in Merino and Idalia, Colorado. All leaf tissue was freeze dried using a lyophilizer (Labconco, Kansas City, MO) prior to DNA extraction.

### Growth conditions and tetrahydrocannabinol quantity measurements

Four seeds of each accession were planted in 3″ × 3″ pots in wet ProMix soil. The pots were kept damp through the germination window and then plants were watered as needed. General Hydroponics (FloraGro, FloraMicro, and FloraBloom) fertilizer was applied 2–3 times in the first 2 months of growth to encourage vegetative growth. No further fertilizing events occurred. Flower tissue samples were harvested from 41 IPK accessions once the plants reached maturity and were oven dried for 2 weeks. No flower tissue samples were harvested from the 20 industrial hemp cultivars.

To assess the quantity of the cannabinoid tetrahydrocannabinol (THC), within harvested flower tissue, each plant’s dried flower biomass was weighed out into 1-g aliquots and stored in separate 50-ml conical centrifuge tubes. These 1-g flower aliquots were then delivered to Botanicor Laboratories in Denver, CO, for standard THC quantity analysis (https://www.botanacor.com). In short, Botanacor Laboratories assess flower samples for THC quantity using Agilent HPLC-DAD instruments. We used previously published data on THC % by mass for 30 accessions from Campbell *et al.* (2019) and Vergara *et al.* (2019). In total, we used THC % by mass for 71 *C. sativa* accessions for which we also had WGS data.

### DNA extraction and whole genome shotgun sequencing

DNA was extracted from plant leaf tissue using the Qiagen DNeasy Plant Mini Kit (Valencia, CA, USA). Extracted DNA was then quantified using a Qubit Fluorometer (ThermoFisher Scientific). Leaf tissue from the 20 European industrial hemp accessions was paired-end whole genome sequenced (2× 150 base pairs) at the Duke University Center for Genomic and Computational Biology using an Illumina HiSeq 4000. Leaf tissue from the 51 IPK accessions was paired-end whole genome sequenced (2× 150 base pairs) at the University of Colorado Anschutz Medical Campus using an Illumina NovaSeq. Sequencing efforts aimed for 4× to 7× coverage of the *C. sativa* genome.

### Genotyping of samples

In addition to our WGS data, 55 publicly available *C. sativa* WGS datasets representing marijuana, industrial hemp and feral *C. sativa* accessions were obtained from Lynch *et al.* (2016). Raw sequence data were initially parsed with FastQC (Andrews 2010, version 0.11.8) to assess read quality and adapter contamination. Trimmomatic (Bolger *et al.* 2014, version 0.39) was then used with default parameters to remove low quality reads and any adapter contamination identified in the FastQC report. The trimmed sequence reads were then aligned to version 2 of the CS10 reference genome (Grassa *et al.* 2021, GenBank assembly accession ID = GCF_900626175.2) using BWA-MEM with default settings (Li 2013, version 0.7.17). Samtools (Li *et al.* 2009, version 1.9) was then used to sort sequence alignment files, mark duplicate reads, keep only properly paired reads, and remove reads with a mapping quality less than 10. BCFtools (Narasimhan *et al.* 2016, version 1.9) was then used with default parameters to identify genetic variants using both the “mpileup” and “call” functions to produce a single variant call file (VCF) for all samples. In total, we genotyped 190 samples for our analyses.

VCFtools (Danecek *et al.* 2011, version 0.1.16) was then used to filter the VCF to contain bi-allelic single-nucleotide polymorphisms (SNPs) that possessed a genotyping rate of ≥80%, quality ≥30, minimum mean read depth ≥2, and minor allele frequency of ≥5%. In addition, to reduce the impact of erroneous heterozygous genotype calls due to the misalignment of paralog and repetitive element reads, we used VCFtools to remove loci in the VCF that had a heterozygous genotype call rate greater than 50% across the 190 samples. The final VCF contained 8,474,449 SNPs.

### Statistical analyses

#### Population structure, cannabinoid correlations, segregating sites, and Tajima’s *D*

A genome-wide principal component analysis (PCA) was conducted using the R (R Core Team 2019, version 3.6.0) package “SNPRelate” (Zheng *et al.* 2012, version 1.18.0). Genetic clustering of samples observed from plotting principal component 1 (PC1) against principal component 2 (PC2) was used to identify subpopulations for downstream analyses. *C. sativa* samples with known source countries were assigned population names based on geographic regions that broadly covered their source and surrounding countries. Thus, we designated “European” (European neighborhood countries), “Asian” (China and North Korea), “US feral” (feral accessions from Colorado, Kansas, and Nebraska), and “South American” (Argentina) as our geographic populations. Marijuana samples (as described in Lynch *et al.* 2016) were classified as “Marijuana” because no distinct clustering based on geography was observed for these samples. Since many of the IPK accessions were not labeled with accurate collection site information, these samples were classified as “IPK unknown.”

To further examine population structure, we used ADMIXTURE (Alexander *et al.* 2009, version 1.3.0) to estimate individual genetic ancestries. The appropriate number of genetic clusters (*K*) was determined using the cross-validation option for *K* values 1 − 10. The *K* value with the lowest cross-validation error was then used for subsequent analyses. After identifying the appropriate *K*, the output “.Q” file from ADMIXTURE was input into the R package “pophelper” (Francis 2017, version 2.2.9) for the visualization of individual ancestry.

Genetic divergence between subpopulations was estimated using VCFtools to calculate the mean Weir and Cockerham fixation index ($\bar{F}$_ST_) using both a pairwise and sliding window approach with 20,000-base pair window sizes (Weir and Cockerham 1984). Sliding window $\bar{F}$_ST_ was visualized using the R package “qqman” (Turner 2018, version 0.1.4).

Means of total potential THC quantities were calculated for each accession with available data using the R package “emmeans” (Lenth *et al.* 2018, version 1.3.5.1). Correlations between accession mean total potential THC quantity and PC1 were then tested using the base R function cor.test() to obtain the Spearman’s rank correlation coefficient (ρ).

Under the out of Asia hypothesis, we would predict deeper coalescence events and thus a greater proportion of segregating sites in the Asian subpopulation. To test the hypothesis that Asian accessions represent the ancestral *C. sativa* population, we performed an analysis to compare the proportion of segregating sites across single-copy orthologs (SCOs) between the Asian, European, US feral, and Marijuana subpopulations. To accomplish this, we first identified 7,540 SCOs between *Ziziphus jujube* (Huang *et al.* 2016), *Parasponia andersonii* (van Velzen *et al.* 2018), and *C. sativa* using OrthoFinder (Emms and Kelly 2019, version 2.2.6) with default parameters. Next, because our Asian population contained 8 samples, we randomly selected 8 samples from the European, US feral, and Marijuana subpopulations to use for comparative analyses of segregating sites. Using the SCO gene coordinates contained in the CS10 gff file, we extracted the VCF loci within the SCO gene intervals for each subpopulation. The R package “PopGenome” (Pfeifer *et al*. 2014, version 2.7.5) was then used to count the number of segregating sites within each SCO interval per subpopulation. A 1-way ANOVA was then performed to compare the mean proportion of segregating sites (per 1 kb) between subpopulations. Tukey-adjusted pairwise comparisons were then used to identify which subpopulations exhibited significantly different proportions of segregating sites. To estimate subpopulation demography, Tajima’s *D* (Tajima 1989) was also calculated for each SCO interval using the R package “PopGenome.” A 1-way ANOVA was then performed to compare the mean Tajima’s *D* between subpopulations. Tukey-adjusted pairwise comparisons were then used to identify which subpopulations exhibited significantly different mean Tajima’s *D* values.

IPK samples without source country information and the 2 South American samples were not included for statistical analyses beyond our PCA, ADMIXTURE, cannabinoid correlations. Asian samples were not included for statistical analyses beyond PCA, ADMIXTURE, THC correlation, proportion of segregating sites, and mean Tajima’s *D* analyses due to small subpopulation sample size.

#### Identification of loci under balancing selection

To identify loci under balancing selection, we first used the R package “PopGenome” to calculate Tajima’s *D* for each SCO interval using all samples within the US feral, European, and marijuana subpopulations separately. We then extracted the loci within SCO interval that possessed a Tajima’s *D* value greater than or equal to the 99th percentile Tajima’s *D* of all SCO intervals within each subpopulation. Counts of site heterozygosity per locus were then calculated and averaged over each SCO interval. Last, SCO intervals that possessed a mean site heterozygosity count greater than or equal to the 70th percentile mean heterozygosity count of all SCO intervals included in the analysis were considered as genes under balancing selection. Genes under balancing selection were then investigated for their annotation and subsequent interpretation. In addition, since many genes lacked detailed annotation, we used the TAIR implementation of basic local alignment search tool (BLASTP) to identify the best homolog match in *Arabidopsis thaliana*.

#### Identification of derived alleles under positive selection

Using the SCOs identified between *Z. jujube*, *P. andersonii*, and *C. sativa*, we estimated the ancestral and derived allelic states within the European, US feral, and Marijuana subpopulations using the methods described in Price *et al.* (2018). For this analysis, we filtered our VCF to contain the 40 Marijuana samples, 40 randomly selected European samples, and all 19 US feral samples. In addition, only loci with a 100% genotyping rate across samples were used in our analyses to avoid frequency bias due to missing genotype information. For downstream analyses of positive selection, we focused only on identifying nonsynonymous derived alleles.

Three separate unfolded joint site frequency spectra were plotted to estimate derived allelic variation between US feral, European, and Marijuana subpopulations. Derived alleles showing evidence for local positive selection (between groups) were identified based on those with nonsynonymous derived allele frequencies ≥70% in 1 group and ≤30% in the other 2 groups. The Fisher’s exact test base R function was then used to confirm significant allele frequency–population associations. Genes containing derived alleles under positive selection were then investigated for their annotation and subsequent interpretation. We then used the TAIR implementation of BLASTP to identify the best homolog match in *A. thaliana*.

Derived alleles showing evidence for species-wide positive selection were identified based on those with nonsynonymous derived allele frequencies greater than 70% across all 3 groups. To broadly describe the function of all genes under species-wide positive selection, we performed a GO analysis for biological processes, molecular function, and cellular component using the locus names for the top *A. thaliana* homolog matches from BLAST in conjunction with the GO Term Enrichment analysis tool implemented in TAIR using the Fisher’s exact test method with a FDR *P*-value cutoff of 0.05.

## Results

### Population structure, correlations, segregating sites, and mean Tajima’s D

**Fig. 1.**
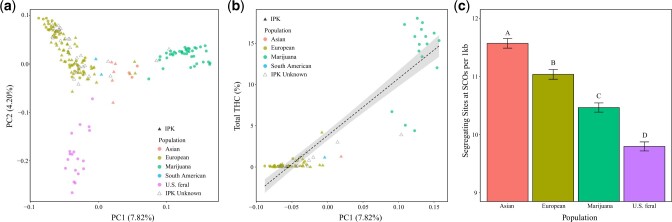
The population structure of *C. sativa*. a) Genome-wide PCA showing clustering and classification of samples. Color of points indicates subpopulation assignment while triangles indicate samples obtained from the Institute of Plant Genetics. b) Correlation between PC1 and mean % total THC for 71 accessions. The black line indicates the regression slope while the gray surrounding area indicates the 95% confidence interval. c) Bar plots showing the comparison of the mean (± standard error) proportion of segregating sites at SCOs. Letters above bars indicate significance of Tukey-adjusted pairwise comparisons such that subpopulations with different letters possess significantly different proportions of segregating sites.

Our genome-wide PCA of the ∼8.5 million SNPs identified evidence for subpopulation structure across the 190 samples. PC1 with PC2 explained 7.82% and 4.20% of the genetic variance, respectively (Fig. 1a). Marijuana samples largely formed a single cluster while *C. sativa* from Europe, Asia, and the United States generally clustered based on geography (Fig. 1a). Two WGS samples sourced from South America representing 1 accession (IPK CAN 51) clustered with European samples while 3 WGS samples representing 2 accessions sourced from Europe (IPK CAN 37 and IPK CAN 58) clustered near Asian *C. sativa*. IPK accessions with unknown source country information mostly clustered with European and Asian samples except for IPK CAN 36, which clustered near the Marijuana group.

ADMIXTURE tests of cross-validation error for *K* values 1–10 indicated *K* = 6 as the best choice of cluster value (Supplementary Fig. 1). Genetic distinctions between samples that broadly resembled their clustering patterns in the PCA were observed (Supplementary Fig. 2). Marijuana samples largely made-up genetic cluster 4. European *C. sativa* were mostly split between genetic clusters 2 and 6. Genetic cluster 5 mostly consisted of US feral plants. All 6 genetic clusters were represented within Asian *C. sativa* with the most predominant being 3 and 4. Most IPK samples of unknown origin and the 2 samples from South America showed evidence for belonging to one of the 2 clusters associated with European accessions.

Our ANOVA comparing the mean proportion of segregating sites across SCOs identified a significant difference (*P* < 0.05) between subpopulations. Post hoc pairwise comparison tests found that each subpopulation included in the analysis significantly differed (*P* < 0.05) in their proportion of segregating sites. The US feral subpopulation exhibited the lowest proportion of segregating sites while the Asian subpopulation exhibited the highest proportion of segregating sites (Fig. 1c), consistent with a deeper coalescent time for ancestral subpopulations.

Our ANOVA comparing the mean Tajima’s *D* across SCOs identified a significant difference (*P* < 0.05) between subpopulations as well. The mean Tajima’s *D* value for all subpopulations were positive. Post hoc pairwise comparison tests found that the US feral and marijuana subpopulations did not significantly differ (*P* > 0.05) from each other and possessed the greatest mean Tajima’s *D* values (Supplementary Fig. 3). The US feral and marijuana subpopulations did however significantly differ (*P* < 0.05) from the European and Asian subpopulations. The Asian subpopulation possessed the lowest mean Tajima’s *D* value and significantly differed (*P* < 0.05) from the European subpopulation.

A significant positive correlation (*P* < 2.2e−16, ρ = 0.80) was observed between % total potential THC (mg/g) and PC1 (Fig. 1b). Samples exhibiting more positive PC1 eigenvalues (*i.e.*, more closely related to Marijuana samples) tended to produce more THC compared to samples with more negative eigenvalues.

### $\bar{F}$ _ST_

**Fig. 2.**
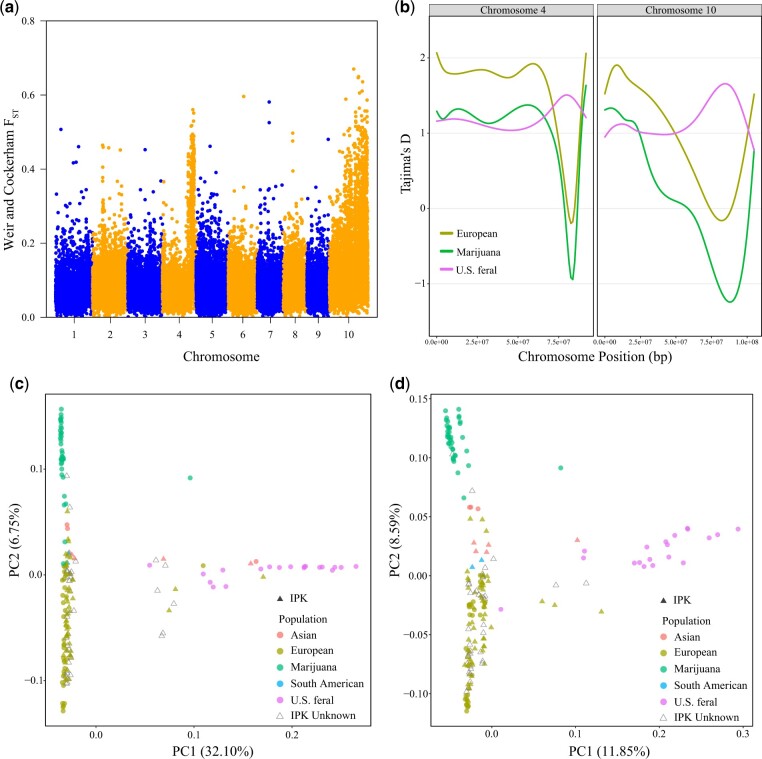
Subpopulation divergence on chromosomes 4 and 10. a) Sliding window $\bar{F}$_ST_ analysis comparing allelic diversity between Marijuana, European and US feral subpopulations. *Y*-axis points indicate $\bar{F}$_ST_ scores across 20,000 base pairs. b) LOWESS plots showing Tajima’s *D* of SCOs plotting against physical positions of chromosomes 4 and 10 (base pairs). Differently colored lines indicate Tajima’s *D* value for different subpopulations. c) Plot of the first 2 principal components of PCA on all SNPs between 77 and 88 Mb of chromosome 4. d) Plot of the first 2 principal components of PCA on all SNPs identified on chromosome 10.

Our pairwise $\bar{F}$_ST_ analysis (Supplementary Table 1) indicated that overall, all the *C. sativa* subpopulations are closely related with the most divergent subpopulations being the US feral and Marijuana samples ($\bar{F}$_ST_ = 0.116). Sliding window $\bar{F}$_ST_ analyses (Fig. 2a) revealed broad genomic regions on chromosomes 4 and 10 that exhibited numerous high $\bar{F}$_ST_ windows. Plotting a locally weighted scatterplot smoothing (LOWESS) of Tajima’s *D* values along chromosome position for chromosomes 4 and 10 revealed that in these high $\bar{F}$_ST_ regions, the US feral subpopulation exhibited positive values while the European and Marijuana subpopulations exhibited negative values (Fig. 2b). Independent PCA analysis of chromosomes 4 and 10 identified distant clustering of US feral samples from all other subpopulation’s samples (Fig. 2, c and d).

### Loci under balancing selection

Of the 75 SCOs possessing mean Tajima’s *D* values greater than or equal to the top 1% in each subpopulation, 23 of these SCOs per subpopulation possessed a mean site heterozygosity greater than or equal to the top 30% of each subpopulation’s respective mean site heterozygosity values (Supplementary Table 2). Thus, these 23 genes per subpopulation were considered to be under balancing selection.

In the US feral subpopulation, the annotation of 6 genes (GenBank peptide accessions: XP_030484994.1, XP_030484921.1, XP_030504444.1, XP_030511229.1, XP_030509614.1, and XP_030486224.1) under balancing selection indicated their involvement in response to numerous biotic and abiotic stresses such as wounding, pathogen, oxidative, salt, and osmotic stress. In the European subpopulation, 4 genes (XP_030510979.1, XP_030482503.1, XP_030478550.1, and XP_030499670.1) under balancing selection were related by their role in maintaining essential chloroplast processes such as: chloroplast gene expression, chloroplast movement in response to light, protein transport into the chloroplast, and thylakoid architecture. The marijuana subpopulation also possessed 4 genes (XP_030510979.1, XP_030480662.1, XP_030493766.1, and XP_030480127.1) under balancing selection that were related by their roles in maintaining essential chloroplast function such as chloroplast gene expression, iron transport into the chloroplast, and chloroplast development during the embryonic stage. Across all these subpopulations 1 gene, XP_030510979.1, was constitutively under balancing selection. This gene’s annotation is *transcription termination factor MTERF9* (*MTERF9*), a chloroplast transcription termination factor that has also shown evidence for being involved in plant response to abiotic stress in *A. thaliana* (Robles *et al.* 2015).

### Derived alleles under positive selection

**Fig. 3.**
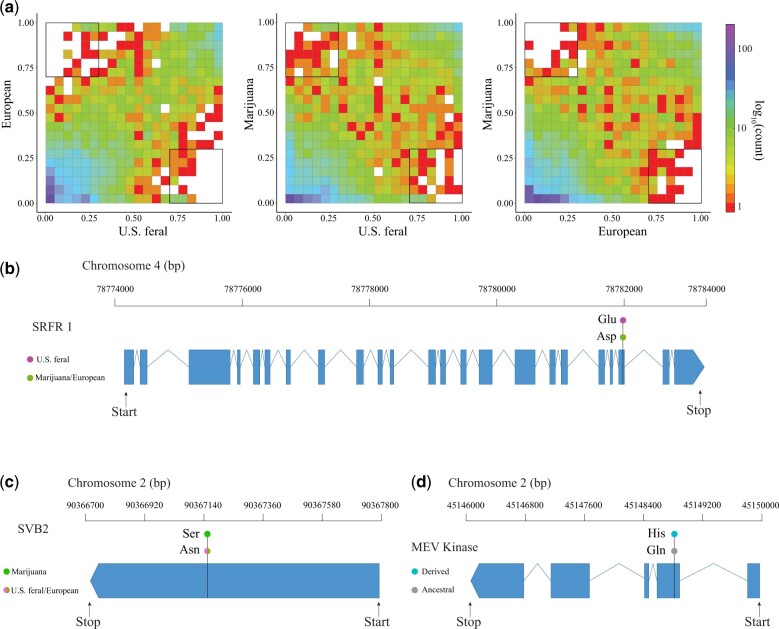
Positive selection in *C. sativa*. a) Derived joint site frequency spectrums showing the derived nonsynonymous allelic diversity between US feral, European, and Marijuana subpopulations. *X* and *Y* axes indicate the frequency of alleles within each bin (squares). The number of derived alleles with each frequency bin is color coded according to the logarithmic scale. The bolded black squares indicate derived alleles at ≥70% frequency. b) *SRFR1* gene model showing the 3 letter codes for the most frequent amino acid substitution within each subpopulation. c) *SVB2* gene model showing the 3 letter codes for the most frequent amino acid substitution within each subpopulation. d) Gene model for *MEV kinase* showing the 3 letter codes for the most frequent amino acid substitution under species-wide positive selection.

Our analysis of the nonsynonymous derived allele frequency spectrum identified 5,143 derived alleles distributed across 2,205 SCOs (Supplementary Table 3). The plotted joint frequency spectra revealed patterns of rare and common derived alleles across the US feral, European, and Marijuana subpopulations while also showing derived alleles at high frequency in 1 group and low frequency in another group (Fig. 3a). Between the 3 *C. sativa* groups, we identified 43, 19, and 7 derived alleles in the US feral, Marijuana, and European samples, respectively, that fit our criteria for subpopulation positive selection (Supplementary Table 4). Fisher’s exact test at each of these loci confirmed significant (*P* < 0.05) allele frequency to subpopulation associations.

Of the genes containing derived alleles showing evidence for subpopulation positive selection, we identified 1 gene potentially contributing to local adaptation in the US feral group whose gene annotation was *suppressor of RPS4-RLD 1* (*SRFR1*, CS_10 gene ID = XP_030499563.1), a gene that has been shown to be involved in defense response to pathogens in *A. thaliana* (Kim *et al.* 2009). We identified interesting genes potentially contributing to local adaptation in the European and Marijuana subpopulations based on their best *A. thaliana* homolog match. In the European subpopulation, the best *A. thaliana* homolog match for one of these genes was AT1G18740/*BYPASS1-LIKE* (annotated as XP_030493166.1 in CS_10), a gene which has been shown to be involved in freezing tolerance (Chen *et al.* 2019). In the Marijuana subpopulation, the best *A. thaliana* homolog match for 1 gene was AT1G09310/*SMALLER TRICHOMES WITH VARIABLE BRANCHES 2* (*SVB2*, annotated as XP_030506485.1 in CS_10), a gene that has been shown to regulate trichome formation (Hussain *et al.* 2021).

At the species-wide level, we identified 439 derived alleles distributed across 349 genes that fit our criteria for species-wide positive selection (Supplementary Table 4). These 439 derived alleles were evenly distributed throughout the *C. sativa* genome (Supplementary Fig. 4). Using the GO Term Enrichment tool implemented in TAIR, we identified 25, 14, and 4 GO terms for biological processes, cellular component, and molecular function, respectively, that were significantly enriched (FDR *P* < 0.05, Supplementary Tables 5–7). The GO terms with the greatest fold change for biological processes, cellular component, and molecular function were chloroplast RNA processing, Golgi cisterna, and zinc ion binding, respectively. Of the genes containing derived alleles showing evidence for species-wide positive selection, one was particularly interesting. This gene was annotated as *mevalonate kinase* (*MEV kinase*), which functions to phosphorylate mevalonate into mevalonate-5-phosphate; a key step in sesquiterpene biosynthesis via the mevalonic acid pathway (Bergman *et al.* 2019).

## Discussion

Whether or not the out of Asia hypothesis for *C. sativa*’s origin is supported remains a central question when studying the evolution of this species. Recently, Ren *et al.* (2021) found evidence supporting the long-held hypothesis that Asian accessions represent the ancestral population of *C. sativa*. Interestingly, the central PCA clustering of the Asian subpopulation we find suggests that this subpopulation shares about equal allelic variation with all other subpopulations (Fig. 1a). If the Asian subpopulation is ancestral, we would predict deeper coalescent events and thus a greater proportion of segregating sites in the Asian subpopulation. After comparing the proportion of segregating sites at SCOs between subpopulations, our results further support an out of Asia origin of *C. sativa* by showing that the Asian subpopulation exhibits a significantly greater (*P* < 0.05) proportion than all other subpopulations (Fig. 1c). In addition, the significantly greater proportion of segregating sites present in the Asian subpopulation suggests a greater effective population size. A greater Asian effective population size was supported by our comparisons of mean Tajima’s *D*, which suggested that all other subpopulations have undergone a recent contraction without time for expansion hence their more positive mean Tajima’s *D* (Supplementary Fig. 3). Thus, we predict deeper coalescent events in the Asian subpopulation, which is supported by their greater proportion of segregating sites across 7,540 SCOs.

### Population structure

In contrast to Marijuana, the European, and Asian subpopulations generally exhibited comparatively greater genomic variation (Fig. 1, a and c). The broad PCA clustering and 2 dominant ancestry likelihoods (2 and 6) of European accessions may reflect subtle genetic changes that occurred within this subpopulation through selective breeding of traits desired for domestication. Interestingly, the population structure of the US feral samples suggests that while this subpopulation likely shares a recent common ancestor with European hemp varieties, it has since undergone significant genomic change, which we hypothesize reflects natural selection against the domestication traits artificially selected for in their ancestors. Overall, IPK accessions with known source country information clustered with other samples from similar regions such as Europe and Asia (Fig. 1a). Except for the IPK accession CAN 36, IPK accessions without known source country information also clustered with samples sourced from Europe and Asia (Fig. 1a). Future studies should make efforts to collect and sequence additional georeferenced accessions across Asia and Africa.

Recent studies have made use of next-generation sequencing methods to describe the population structure of *C. sativa* (Sawler *et al.* 2015; Lynch *et al.* 2016; Soorni *et al.* 2017; Ren *et al.* 2021). A common finding of these prior studies is a clear genetic distinction between Marijuana and non-Marijuana type accessions. In addition, Busta *et al.* (2022) recently analyzed the population structure of feral Nebraskan *C. sativa* and found that they were more closely related to hemp type cultivars than marijuana type cultivars. While we did find evidence to support these prior finding (Fig. 1, a and b), our study also made large-scale efforts to generate new WGS data of feral accessions in the United States (Colorado), European industrial hemp varieties, and accessions from the IPK seed bank. By including new WGS data of these previously unexamined accessions, in addition to publicly available data, our analyses provide results that expand the current knowledge of *C. sativa* population genomics.

### Subpopulation divergence

Consistent with our pairwise $\bar{F}$_ST_ results, numerous studies have previously documented relatively low population divergence between *C. sativa* subpopulations (Sawler *et al.* 2015; Lynch *et al.* 2016; Ren *et al.* 2021). The higher pairwise $\bar{F}$_ST_ scores of the feral US vs. the European and Marijuana subpopulation we found (Supplementary Table 1), however, is potentially a result from the broad high $\bar{F}$_ST_ genomic regions on chromosomes 4 and 10 (Fig. 2a). In addition, in contrast to the European and marijuana subpopulations, the US feral subpopulation exhibited comparatively greater genetic variation in these regions as well based on our Tajima’s *D* analyses (Fig. 2b). Due to their substantial size, we hypothesize that these broad high $\bar{F}$_ST_ regions may be the result of chromosomal inversions maintained at high frequency in the feral US subpopulation. Chromosomal inversions have been previously associated with broad high $\bar{F}$_ST_ regions in other plant species such as sunflower (Todesco *et al.* 2020). Future studies should make efforts to assemble a chromosome scale reference genome representative of these US feral accessions to validate any potential inversions in this subpopulation.

### Balancing selection

Our Tajima’s *D* and mean heterozygosity analyses together identified numerous genes under balancing selection within the US feral, European, and marijuana subpopulations. While collectively all the genes we identified under balancing selection spanned a diversity of functions, subsets of genes within each subpopulation were broadly related by their similar annotation. In the US feral subpopulation, 6 genes involved in plant response to various biotic and abiotic stresses were identified to be under balancing selection. This is interesting because a consequence of domestication is the loss of adaptive genetic variation for survival in nature to favor fitness for human purpose (Brown 2010). The US feral subpopulations have been evolving under natural selection, which has likely favored survival traits that were selected against in their domesticated ancestors. Thus, we hypothesize that the maintenance of genetic variation at these stress response loci in the US feral subpopulation reflects selective pressure from a loss of domestication syndrome that selects for traits, which allow for adaptation to novel perturbations in this subpopulation’s wild landscape via heterozygote advantage.

In both the European and marijuana subpopulations, 4 genes broadly involved in the maintenance of essential chloroplast functions were identified to be under balancing selection. It is interesting that these 2 subpopulations, which contain many domesticated genotypes, both exhibit evidence for balancing selection at functionally related (yet distinct) loci despite being domesticated for different traits. While it remains unclear as to why, we hypothesize that, in contrast to the US feral subpopulation, the maintenance of genetic variation at genes broadly related to chloroplast function in both the European and marijuana subpopulations reflects selection for traits favored by domestication that exhibit a heterozygote advantage.

Interestingly, the gene *MTERF9* met our criteria for balancing selection across all 3 subpopulations. In *A. thaliana*, mutagenesis of *MTERF9* has been shown to cause altered chloroplast gene expression, defective chloroplast development, and altered plant response to abiotic stresses (Robles *et al.* 2015). Our finding that genetic variation is maintained at *MTERF9* across divergent subpopulations of *C. sativa* is intriguing and suggests a species-wide heterozygous advantage at this gene. Similar findings of genomic regions with species-wide maintenance of heterozygosity have been observed in other domesticated plant species such as maize where it is hypothesized that a homozygous state of these loci produces a deleterious phenotype via inbreeding depression (Brandenburg *et al.* 2017; Liu *et al.* 2018). The maintenance of genetic variation at *MTERF9* across *C. sativa* we find may indicate a similar phenomenon as predicted in maize where a homozygous state of this gene results in a species-wide cost to fitness given its broadly important role in chloroplast gene expression, development, and plant response to abiotic stress. Thus, we hypothesize that the genetic variation maintained at *MTERF9* in *C. sativa* reflects species-wide balancing selection for heterozygote advantage.

### Positive selection

Broadly, our analysis of the derived nonsynonymous allele frequency spectra identified results consistent with population genetics theory wherein we observed both rare and fixed alleles in each subpopulation (Nielsen 2005). Furthermore, the results obtained from the joint derived allele frequency spectra suggest that while overall most high-frequency-derived alleles are common to the US feral, European, and Marijuana subpopulations, there are some highly diverged loci across the genome, which serve as the basis of higher within-group similarity (Fig. 3a).

Interestingly, the US feral *C. sativa* contained the greatest proportion of high-frequency-derived alleles among the 3 subpopulations. This finding, in conjunction with the results from our other analyses, suggests that the feral US subpopulation has undergone significant genetic change which we hypothesize indicates local adaptation to new climates, biotic interactions, and relaxed selection on traits favored by domestication. Indeed, 1 gene containing a high-frequency-derived allele in the US feral group, which may contribute to local adaptation to novel pathogens was *SRFR1* (Fig. 3b), a gene for which numerous studies have documented its role in defense against pathogenic bacteria in *A. thaliana* (Kim *et al.* 2009, 2010; Li *et al.* 2010). Positive selection on *SRFR1* is also interesting because it suggests that in addition to maintenance of genetic variation at loci associated with stress response, adaptation to natural environments during feralization occurs through fixation of newly emerged mutations.

By identifying the best homolog matches in *A. thaliana* for unannotated genes with high-frequency-derived alleles, we were also able to discover candidate genes that potentially underlie local adaptation in the European and Marijuana subpopulations. In the European subpopulation, the gene annotated as XP_030493166.1 was found to be under positive selection. The best homolog match for this gene was AT1G18740/*BYPASS1-LIKE*, a gene that has been shown to confer freezing tolerance in *A. thaliana* via the C—repeat binding factor pathway (Chen *et al.* 2019). We hypothesize that the positive selection on the derived allele in XP_030493166.1 reflects selection in domesticated hemp cultivars for increased fitness in lower temperatures experienced across European climates.

In the Marijuana subpopulation, our finding that AT1G09310/*SVB2* was the best homolog match for an unannotated gene (XP_030506485.1) containing a derived allele under positive selection was particularly interesting. Variation in *SVB2* has been shown to regulate trichome number in *A. thaliana* (Hussain *et al.* 2021). Positive selection on this gene in Marijuana is interesting because the primary sought after product from cultivation of marijuana is THC—a psychoactive cannabinoid that is largely produced in the glandular trichomes of female flowers via the polyketide pathway (Kovalchuk *et al.* 2020). In addition, it has been shown in *C. sativa* that yield of cannabinoids is positively correlated with the number of capitate glandular trichomes present on bracts (Turner *et al.* 1981). The association between the functionality of *SVB2* and the primary cultivation of marijuana for THC suggests that the positive selection we observe on the derived allele in *SVB2* is driven by humans to develop domesticated cultivars with a greater number of glandular trichomes that subsequently produce greater quantities of THC. Future studies will need to utilize functional genetics to identify potential phenotypes associated with the derived alleles we identified in *SRFR1*, *BYPASS1-LIKE*, and *SVB2*.

Our efforts to identify positively selected derived alleles at the species-wide level yielded genes that spanned a diversity of significantly enriched ontologies (Supplementary Tables 5–7). Interestingly, 1 gene with a derived allele positively selected at the species-wide level was *MEV kinase*, which functions to phosphorylate mevalonate into mevalonate-5-phosphate (Bergman *et al.* 2019); a key step in sesquiterpene biosynthesis via the mevalonic acid pathway (Kovalchuk *et al.* 2020). While sesquiterpenes, such as beta caryophyllene, are known for their natural role in plant defense response (Rasmann *et al.* 2005), they are also of great interest to the *C. sativa* science community because of their use as food additives and potential for use in human medicine (Nuutinen 2018). The broad application of sesquiterpenes in both nature and humans suggests 2 possible hypotheses for why the derived allele in *MEV kinase* has been positively selected at the species-wide level in *C. sativa*. First, we hypothesize that the derived allele was positively selected in the ancestral subpopulation for enhanced resistance to novel pathogens. Alternatively, we hypothesize that the derived allele was indirectly selected for during the early domestication of *C. sativa* in Asia to produce cultivars with sesquiterpene production more well suited for food or medicinal applications (Li 1973). Future studies will need to utilize functional genetics to identify a phenotype associated with the derived allele we found in *MEV kinase* to validate our hypotheses.

### Conclusion

In conclusion, as regulations around the world regarding *C. sativa*’s legality have increasingly relaxed, the volume of genomics studies on this species has steadily grown. This is exciting because it has enabled researchers to use whole genome methods for investigating fundamental population genetic questions about this multifaceted crop’s evolutionary history. While progress has been made in understanding this species’ evolution, several areas require additional attention such as describing the genetic variation present within the multitude of diverse uncharacterized accessions, formal testing of hypotheses regarding this species’ origin, and identification of the loci under selection that genetically distinguish domesticated from feral subpopulations. Our study addresses each of these areas by (1) large-scale WGS of previously unexamined accessions, (2) further supporting the Asian origin of *C. sativa*, and (3) identifying loci under balancing and positive selection that potentially serve as the basis for local adaptation at both the subpopulation and species-wide levels. While further steps remain necessary to: (1) sequence additional accessions, (2) assemble a genome representative of feral US plants, and (3) functionally validate the derived alleles under positive selection identified here, we believe overall that the data generated, and results obtained from our study help to form a better understanding of the evolutionary history in *C. sativa*.

## Supplementary Material

jkac209_Supplemental_Figures

jkac209_Supplemental_Tables

## Data Availability

All raw paired-end fastq files produced from our WGS efforts have been deposited to NCBI’s short read archive under BioProject Accession number: PRJNA866500. Additional data generated in this study are available in Supplementary File 1. Supplementary Fig. 5 shows the individual-derived allele frequency spectrums for the US feral, European, and Marijuana subpopulations. Supplementary Tables 8–11 describe the sliding window *F*_ST_ scores, PCA values, means of total potential THC for IPK accessions, and Tajima’s *D* and segregating sites of the SCO intervals, respectively. The final filtered VCF has been deposited to Dryad and given the DOI link: https://doi.org/10.5061/dryad.rv15dv49q. Supplemental material is available at *G3* online.

## References

[jkac209-B1] Alexander DH , Novembre J, Lange K. Fast model-based estimation of ancestry in unrelated individuals. Genome Res. 2009;19(9):1655–1664. 10.1101/gr.094052.109 19648217 PMC2752134

[jkac209-B3] Andrews S. FastQC. Babraham Bioinformatics; 2010. https://doi.org/citeulike-article-id:11583827. http://www.bioinformatics.babraham.ac.uk/projects/fastqc/

[jkac209-B5] Begun DJ , Holloway AK, Stevens K, Hillier LW, Poh Y-P, Hahn MW, Nista PM, Jones CD, Kern AD, Dewey CN, et al Population genomics: whole-genome analysis of polymorphism and divergence in *Drosophila simulans*. PLoS Biol. 2007;5(11):e310. 10.1371/journal.pbio.0050310 17988176 PMC2062478

[jkac209-B6] Bergman ME , Davis B, Phillips MA. Medically useful plant terpenoids: biosynthesis, occurrence, and mechanism of action. Molecules. 2019;24(21):3961. 10.3390/molecules24213961 31683764 PMC6864776

[jkac209-B7] Bolger AM , Lohse M, Usadel B. Trimmomatic: a flexible trimmer for Illumina sequence data. Bioinformatics. 2014;30(15):2114–2120. 10.1093/bioinformatics/btu170 24695404 PMC4103590

[jkac209-B8] Brandenburg J-T , Mary-Huard T, Rigaill G, Hearne SJ, Corti H, Joets J, Vitte C, Charcosset A, Nicolas SD, Tenaillon MI. Independent introductions and admixtures have contributed to adaptation of European maize and its American counterparts. PLoS Genet. 2017;13(3):e1006666. 10.1371/journal.pgen.1006666 28301472 PMC5373671

[jkac209-B9] Brown AHD. Variation under domestication in plants: 1859 and today. Phil Trans R Soc B. 2010;365(1552):2523–2530. 10.1098/rstb.2010.0006 20643742 PMC2935096

[jkac209-B10] Busta L , Dweikat I, Sato SJ, Qu H, Xue Y, Zhou B, Gan L, Yu B, Clemente TE, Cahoon EB, et al Chemical and genetic variation in feral *Cannabis sativa* populations across the Nebraska climate gradient. Phytochemistry. 2022;200:113206. 10.1016/j.phytochem.2022.113206 35436478

[jkac209-B11] Campbell BJ , Berrada AF, Hudalla C, Amaducci S, McKay JK. Genotype × environment interactions of industrial hemp cultivars highlight diverse responses to environmental factors. Agrosyst Geosci Environ. 2019;2(1):1. 10.2134/age2018.11.0057

[jkac209-B12] Charlesworth B , Charlesworth D. Elements of Evolutionary Genetics. Greenwood Village, CO: Roberts and Company; 2010.

[jkac209-B13] Chen T , Chen J-H, Zhang W, Yang G, Yu L-J, Li D-M, Li B, Sheng H-M, Zhang H, An L-Z. BYPASS1-LIKE, a DUF793 family protein, participates in freezing tolerance Via the CBF pathway in *Arabidopsis*. Front Plant Sci. 2019;10:807. 10.3389/fpls.2019.00807 31297122 PMC6607965

[jkac209-B14] Danecek P , Auton A, Abecasis G, Albers CA, Banks E, DePristo MA, Handsaker RE, Lunter G, Marth GT, Sherry ST, et al; 1000 Genomes Project Analysis Group. The variant call format and VCFtools. Bioinformatics. 2011;27(15):2156–2158. 10.1093/bioinformatics/btr330 21653522 PMC3137218

[jkac209-B15] Darwin C , Kebler L. On the Origin of species by Means of Natural Selection, or, the Preservation of Favoured Races in the Struggle for Life. London: J. Murray; 1859. PMC5184128 30164232

[jkac209-B16] Emms DM , Kelly S. OrthoFinder: phylogenetic orthology inference for comparative genomics. Genome Biol. 2019;20(1). 10.1186/s13059-019-1832-y PMC6857279 31727128

[jkac209-B19] Francis RM. pophelper: an R package and web app to analyse and visualize population structure. Mol Ecol Resour. 2017;17(1):27–32. 10.1111/1755-0998.12509 26850166

[jkac209-B20] Grassa CJ , Weiblen GD, Wenger JP, Dabney C, Poplawski SG, Timothy Motley S, Michael TP, Schwartz CJ. A new *Cannabis* genome assembly associates elevated cannabidiol (CBD) with hemp introgressed into marijuana. New Phytol. 2021;230(4):1665–1679. 10.1111/nph.17243 33521943 PMC8248131

[jkac209-B21] Huang J , Zhang C, Zhao X, Fei Z, Wan K, Zhang Z, Pang X, Yin X, Bai Y, Sun X, et al The jujube genome provides insights into genome evolution and the domestication of sweetness/acidity taste in fruit trees. PLoS Genet. 2016;12(12):e1006433. 10.1371/journal.pgen.1006433 28005948 PMC5179053

[jkac209-B22] Hussain S , Zhang N, Wang W, Ahmed S, Cheng Y, Chen S, Wang X, Wang Y, Hu X, Wang T, et al Involvement of aba responsive svb genes in the regulation of trichome formation in *Arabidopsis*. IJMS. 2021;22(13):6790. 10.3390/ijms22136790 34202673 PMC8268597

[jkac209-B24] Kim SH , Gao F, Bhattacharjee S, Adiasor JA, Nam JC, Gassmann W. The *Arabidopsis* resistance-like gene SNC1 is activated by mutations in SRFR1 and contributes to resistance to the bacterial effector AvrRps4. PLoS Pathog. 2010;6(11):e1001172. 10.1371/journal.ppat.1001172 21079790 PMC2973837

[jkac209-B25] Kim SH , Kwon S, Il, Saha D, Anyanwu NC, Gassmann W. Resistance to the *Pseudomonas syringae* effector hopA1 is governed by the TIR-NBS-LRR Protein rps6 and is enhanced by mutations in srfr1. Plant Physiol. 2009;150(4):1723–1732. 10.1104/pp.109.139238 19525323 PMC2719129

[jkac209-B26] Kovalchuk I , Pellino M, Rigault P, van Velzen R, Ebersbach J, Ashnest JR, Mau M, Schranz ME, Alcorn J, Laprairie RB, et al The genomics of *Cannabis* and its close relatives. Annu Rev Plant Biol. 2020;71(1):713–739. 10.1146/annurev-arplant-081519-040203 32155342

[jkac209-B27] Larson G , Piperno DR, Allaby RG, Purugganan MD, Andersson L, Arroyo-Kalin M, Barton L, Climer Vigueira C, Denham T, Dobney K, et al Current perspectives and the future of domestication studies. Proc Natl Acad Sci USA. 2014;111(17):6139–6146. 10.1073/pnas.1323964111 24757054 PMC4035915

[jkac209-B28] Laursen L. Botany: the cultivation of weed. Nature. 2015;525(7570):S4–S5. 10.1038/525S4a 26398737

[jkac209-B29] Lenth R , Singmann H, Love J, Buerkner P, Herve M. Package ‘emmeans’. Estimated marginal means, aka least-squares means. R Package Version 1.15-15; 2018.

[jkac209-B30] Li H. Aligning sequence reads, clone sequences and assembly contigs with BWA-MEM. 2013;1–3. https://arxiv.org/abs/1303.3997.

[jkac209-B31] Li HL. An archaeological and historical account of cannabis in China. Econ Bot. 1973;28(4):437–448. 10.1007/BF02862859

[jkac209-B32] Li H , Handsaker B, Wysoker A, Fennell T, Ruan J, Homer N, Marth G, Abecasis G, Durbin R; 1000 Genome Project Data Processing Subgroup. The sequence alignment/map format and SAMtools. Bioinformatics. 2009;25(16):2078–2079. 10.1093/bioinformatics/btp352 19505943 PMC2723002

[jkac209-B33] Li Y , Li S, Bi D, Cheng YT, Li X, Zhang Y. SRFR1 negatively regulates plant NB-LRR resistance protein accumulation to prevent autoimmunity. PLoS Pathog. 2010;6(9):e1001111. 10.1371/journal.ppat.1001111 20862316 PMC2940742

[jkac209-B34] Liu N , Liu J, Li W, Pan Q, Liu J, Yang X, Yan J, Xiao Y. Intraspecific variation of residual heterozygosity and its utility for quantitative genetic studies in maize. BMC Plant Biol. 2018;18(1). 10.1186/s12870-018-1287-4 PMC5909218 29673320

[jkac209-B35] Lynch RC , Vergara D, Tittes S, White K, Schwartz CJ, Gibbs MJ, Ruthenburg TC, deCesare K, Land DP, Kane NC. Genomic and chemical diversity in *Cannabis*. Crit Rev Plant Sci. 2016;35(5–6):349–363. 10.1080/07352689.2016.1265363

[jkac209-B36] Mace ES , Tai S, Gilding EK, Li Y, Prentis PJ, Bian L, Campbell BC, Hu W, Innes DJ, Han X, et al Whole-genome sequencing reveals untapped genetic potential in Africa’s indigenous cereal crop sorghum. Nat Commun. 2013;4(1): 10.1038/ncomms3320 PMC3759062 23982223

[jkac209-B40] Narasimhan V , Danecek P, Scally A, Xue Y, Tyler-Smith C, Durbin R. BCFtools/RoH: a hidden Markov model approach for detecting autozygosity from next-generation sequencing data. Bioinformatics. 2016;32(11):1749–1751. 10.1093/bioinformatics/btw044 26826718 PMC4892413

[jkac209-B41] Nielsen R. Molecular signatures of natural selection. Annu Rev Genet. 2005;39(1):197–218. 10.1146/annurev.genet.39.073003.112420 16285858

[jkac209-B42] Nuutinen T. Medicinal properties of terpenes found in *Cannabis sativa* and *Humulus lupulus*. Eur J Med Chem. 2018;157:198–228. 10.1016/j.ejmech.2018.07.076 30096653

[jkac209-B31023325] Pfeifer B , Wittelsbürger U, Ramos-Onsins SE, Lercher MJ. PopGenome: an efficient Swiss army knife for population genomic analyses in R. Mol Biol Evol. 2014;31(7):1929–1936. 24739305 10.1093/molbev/msu136 PMC4069620

[jkac209-B44] Price N , Moyers BT, Lopez L, Lasky JR, Monroe JG, Mullen JL, Oakley CG, Lin J, Ågren J, Schrider DR, et al Combining population genomics and fitness QTLs to identify the genetics of local adaptation in *Arabidopsis thaliana*. Proc Natl Acad Sci U S A. 2018;115(19):5028–5033. 10.1073/pnas.1719998115 29686078 PMC5948977

[jkac209-B45] Qin C , Yu C, Shen Y, Fang X, Chen L, Min J, Cheng J, Zhao S, Xu M, Luo Y, et al Whole-genome sequencing of cultivated and wild peppers provides insights into *Capsicum* domestication and specialization. Proc Natl Acad Sci U S A. 2014;111(14):5135–5140. 10.1073/pnas.1400975111 24591624 PMC3986200

[jkac209-B46] R Core Team . R Foundation for Statistical Computing: Vienna, Austria; 2019.

[jkac209-B47] Rasmann S , Köllner TG, Degenhardt J, Hiltpold I, Toepfer S, Kuhlmann U, Gershenzon J, Turlings TCJ. Recruitment of entomopathogenic nematodes by insect-damaged maize roots. Nature. 2005;434(7034):732–737. 10.1038/nature03451 15815622

[jkac209-B48] Ren G , Zhang X, Li Y, Ridout K, Serrano-Serrano ML, Yang Y, Liu A, Ravikanth G, Nawaz MA, Mumtaz AS, et al Large-scale whole-genome resequencing unravels the domestication history of *Cannabis sativa*. Sci Adv. 2021;7(29): 10.1126/sciadv.abg2286 PMC8284894 34272249

[jkac209-B49] Robles P , Micol JL, Quesada V. Mutations in the plant-conserved MTERF9 alter chloroplast gene expression, development and tolerance to abiotic stress in *Arabidopsis thaliana*. Physiol Plant. 2015;154(2):297–313. 10.1111/ppl.12307 25393651

[jkac209-B51] Sawler J , Stout JM, Gardner KM, Hudson D, Vidmar J, Butler L, Page JE, Myles S. The genetic structure of marijuana and hemp. PLoS One. 2015;10(8):e0133292. 10.1371/journal.pone.0133292 26308334 PMC4550350

[jkac209-B52] Scossa F , Fernie AR. When a crop goes back to the wild: feralization. Trends Plant Sci. 2021;26(6):543–545. 10.1016/j.tplants.2021.02.002 33674174

[jkac209-B54] Soorni A , Fatahi R, Haak DC, Salami SA, Bombarely A. Assessment of genetic diversity and population structure in Iranian *Cannabis* germplasm. Sci Rep. 2017;7(1): 10.1038/s41598-017-15816-5 PMC5688169 29142201

[jkac209-B55] Tajima F. Statistical method for testing the neutral mutation. Genetics. 1989;123(3):585–595. 2513255 10.1093/genetics/123.3.585 PMC1203831

[jkac209-B56] Todesco M , Owens GL, Bercovich N, Légaré J-S, Soudi S, Burge DO, Huang K, Ostevik KL, Drummond EBM, Imerovski I, et al Massive haplotypes underlie ecotypic differentiation in sunflowers. Nature. 2020;584(7822):602–607. 10.1038/s41586-020-2467-6 32641831

[jkac209-B57] Turner JC , Hemphill JK, Mahlberg PG. Interrelationships of glandular trichomes and cannabinoid content. I. Developing pistillate bracts of *Cannabis sativa* L. (Cannabaceae). Bull Narc. 1981;33(2):59–69. 6914206

[jkac209-B58] Turner SD. qqman: an R package for visualizing GWAS results using Q-Q and manhattan plots. JOSS. 2018;3(25):731. 10.21105/joss.00731

[jkac209-B59] van Velzen R , Holmer R, Bu F, Rutten L, van Zeijl A, Liu W, Santuari L, Cao Q, Sharma T, Shen D, et al Comparative genomics of the nonlegume *Parasponia* reveals insights into evolution of nitrogen-fixing rhizobium symbioses. Proc Natl Acad Sci U S A. 2018;115(20). 10.1073/pnas.1721395115 PMC5960304 29717040

[jkac209-B60] Vergara D , Huscher EL, Keepers KG, Givens RM, Cizek CG, Torres A, Gaudino R, Kane NC. Gene copy number is associated with phytochemistry in *Cannabis sativa*. AoB PLANTS. 2019;11(6): 10.1093/aobpla/plz074 PMC6986684 32010439

[jkac209-B61] Weir BS , Cockerham CC. Estimating F-statistics for the analysis of population structure. Evolution. 1984;38(6):1358–1370. 10.1111/j.1558-5646.1984.tb05657.x 28563791

[jkac209-B62] West DP. Hemp and Marijuana: Myths & Realities. North American Industrial Hemp Council; 1998.

[jkac209-B63] Zheng X , Levine D, Shen J, Gogarten SM, Laurie C, Weir BS. A high-performance computing toolset for relatedness and principal component analysis of SNP data. Bioinformatics. 2012;28(24):3326–3328. 10.1093/bioinformatics/bts606 23060615 PMC3519454

