# Supplementary material for: Genome-wide polymorphism and genic selection in feral and domesticated lineages of Cannabis sativa
Source: G3 (Bethesda). 2022 Aug 26;13(2):jkac209. doi: 10.1093/g3journal/jkac209 (PMC9911069; doi:10.1093/g3journal/jkac209)
Supplement: jkac209_Supplemental_Figures [file jkac209_supplemental_figures.pdf]

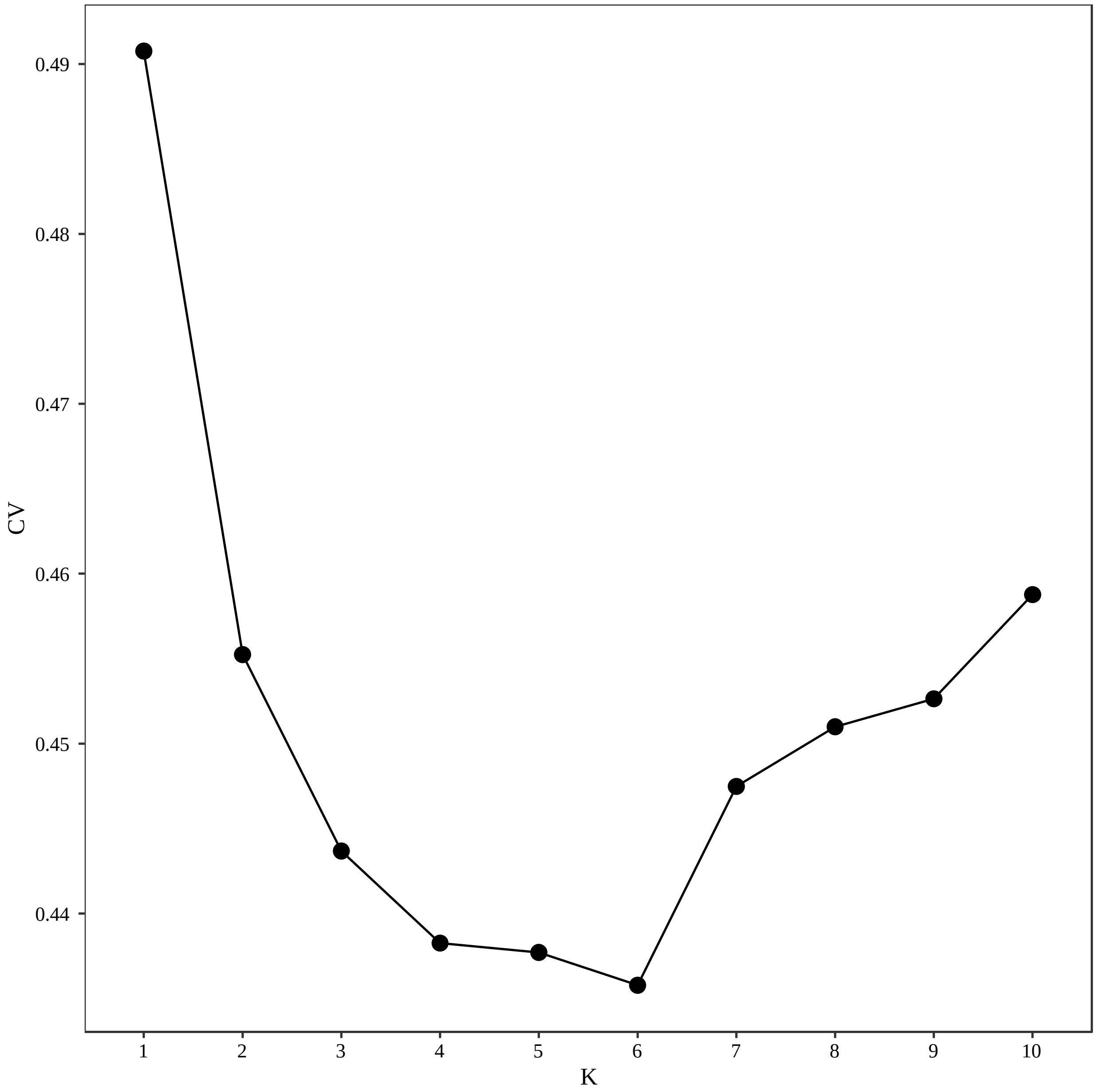

Supplemental Figure 1. Cross validation (CV) errors calculated by ADMIXTURE for K values 1-10. K = 6 exhibited the lowest cross validation error.

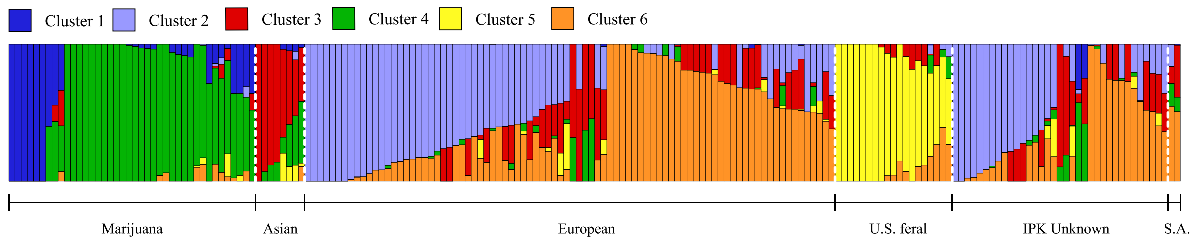

Supplemental Figure 2. Stacked bar plots representing ADMIXTURE results showing individual ancestry likelihoods assuming K = 6.

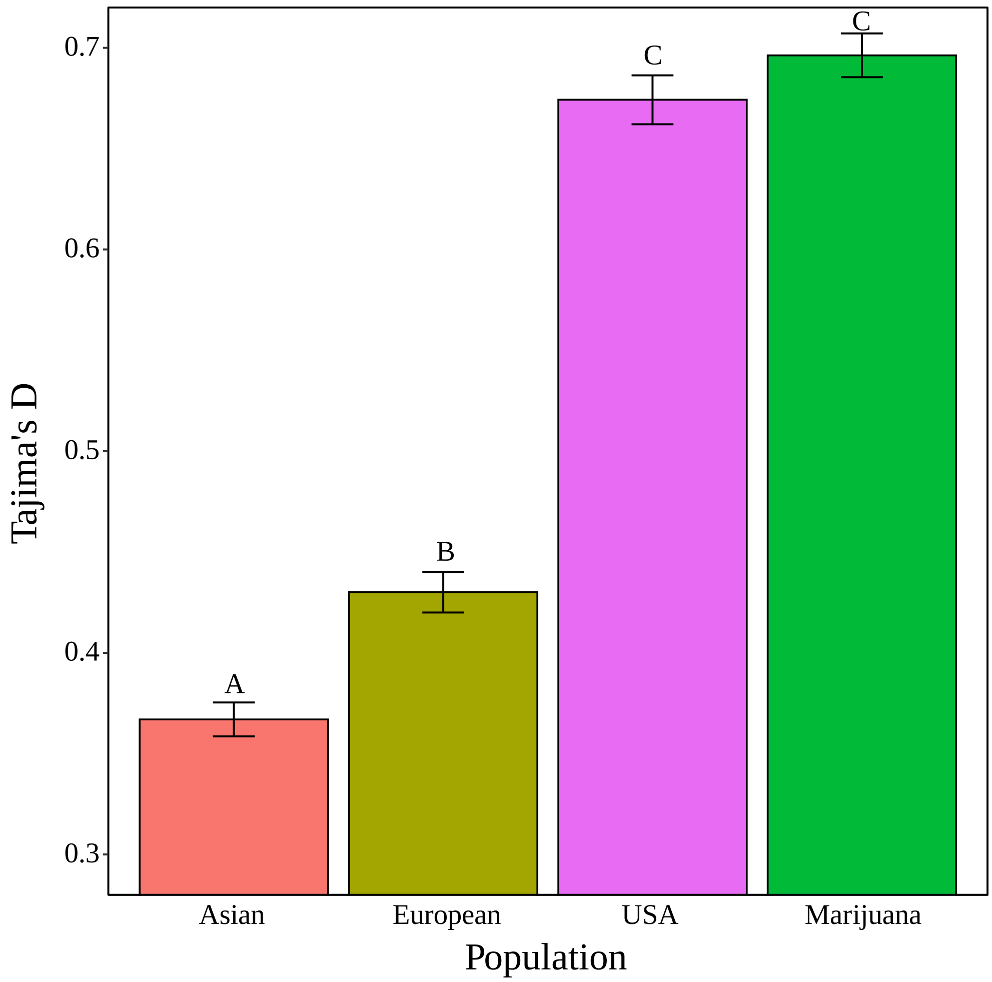

Supplemental Figure 3. Bar plots showing the comparisons of mean Tajima's D (± standard error) across the Asian, European, U.S. feral and Marijuana sub-populations at the 7540 single copy orthologs. Letters above bars indicate significance of Tukey adjusted post-hoc pairwise comparisons such that different letters indicate significant differences ($p < 0.05$).

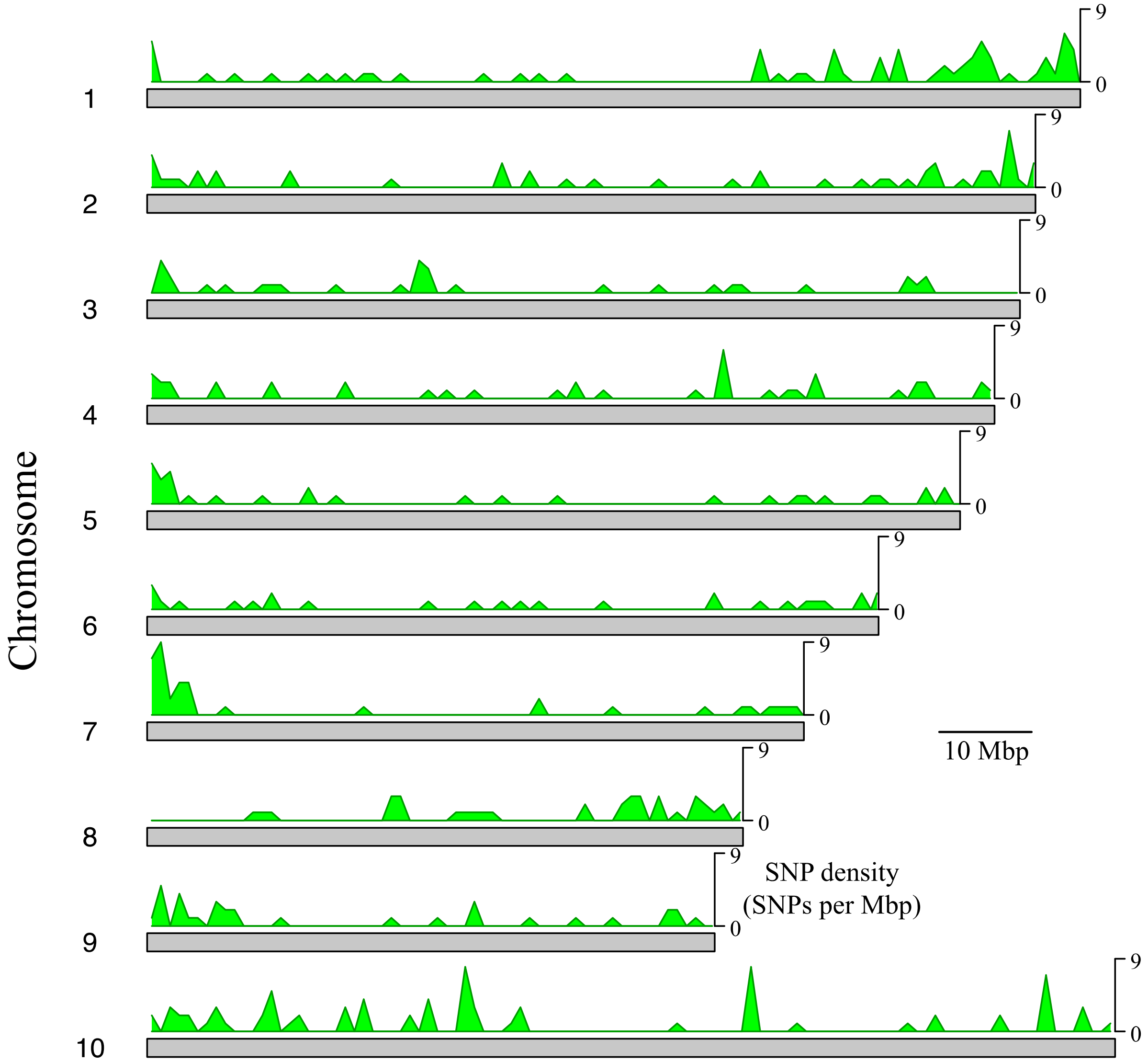

Supplemental Figure 4. SNP density plot depicting the locations of derived alleles showing evidence for species wide selection. Shown are horizontal grey bars representing the ten *C. sativa* chromosomes with local green density plots that depict the number of species wide high frequency derived alleles in 1 megabase intervals.

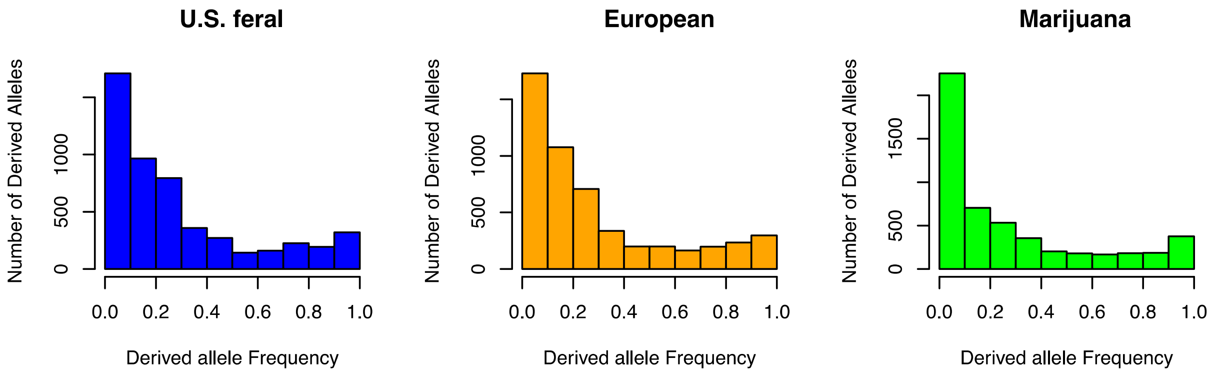

Supplemental Figure 5. Individual derived allele frequency spectrums for the U.S. feral, European and Marijuana sub-populations respectively.
